# Do We Ever Forget?

**Published:** 1854-11

**Authors:** 


From the German of Seguern.

### DO WE EVER FORGET?

One of the most startling and mysterious phenomena of our nature is the sudden revival of the recollection of scenes, events and thoughts which had apparently been long forgotten. In many instances we can explain this by the law of association; but not unfrequently the recollection flashes without warning upon the mind. It is as though we had been gazing out into the blank darkness, which lighted up all at once by a sudden flash, should become a theatre upon which the minutest events of our past life are re-enacted.

Phenomena of this kind, more or less distinctly marked, occur in the experience of every individual, in his ordinary and normal states. But here, as in many other cases, great light is thrown upon the latent capabilities of the mind by its action, when physical disease has induced changes in the conditions which regulate its manifestation. The bodily organs in the healthy state seem to act as checks and as limitations upon the operations of the mind, somewhat as the balance wheel of a watch checks and regulates the uncoiling of the spring. We do not know how rapidly the wheels may be impelled until the check is taken off. The balance wheel makes the watch move in time; and it may be the limitation of the bodily organs only which compel the mind to act in reference to time. A disembodied spirit may have as little to do with time as with space. To all spirits, in their degree, as well as to the Supreme Spirit, one day may, in the literal acceptation of the words, be as a thousand years, and a thousand years as one day; so that in the future life we may continually live over again every portion of our past existence, not piecemeal and fragmentarily, but as an undivided whole; just as the eye takes in a single glance the whole prospect before it, no matter though it be bounded only by the remotest distance from which the farthest ray of light has come which has been casting upward since creation.

Something of this sort has been remarked by those few who have so nearly passed the boundaries between the present and the future life, that they have won a glimpse of that "undiscovered country, from whose bourne," the great dramatist assumes, falsely perhaps, "no traveller returns." De Quincy, the "English opium eater," relates an incident of this kind of a friend who was once at the point of death by drowning. At the moment when she was on the verge of death, she saw her whole life, down to its minute and apparent trivial incidents arrayed before her, as if in a mirror; and at the same time she felt within herself the sudden development of a faculty for comprehending the whole and every part. And he intimates that the possibility of this mighty development is confirmed by experiences of his during that abnormal relation between his spiritual and physical nature which had been induced by the use of opium. Abercrombie relates the case of a boy, who at the age of four years, was rendered insensible by some violence, which fractured his skull. In this state he underwent the operation of trepanning. After recovery, he retained no recollection of the operation, or of the accident which occasioned it. More than ten years after he was seized with a violent fever, during which he became delirious. And now the faint traces made so long ago upon his consciousness —traces so faint that there was no reason to suspect their existence—were brought out under the fierce alchemy of disease, with the utmost distinctness, and he related the occurrence with the utmost minuteness.

One of the most common phenomena in respect to old age, is the re-awakening of the dormant recollections of childhood. Many cases are on record of emigrants who left our German Father land, and have sought a new home in America, at so early an age as to have forgotten their native language; but when, often in the extremest age, they lay upon the bed of death, those long-forgotten words come back to their recollection, and their latest prayers are breathed in the language in which their cradle hymns were sung.*

* We were once personally familiar with a remarkable case of this nature. A clergyman was called to visit an aged man who appeared to be dying. He had been married nearly fifty years, and was surrounded by his wife and children, but in his illness he began to talk to them in an unknown tongue, and could not use the English language, which he had spoken for half a century. It was impossible for him to make himself understood, and as he had led a wicked life, and seemed evidently greatly distressed with his nearness to death, it was painful to witness his unavailing efforts to communicate his thoughts. He was one of the Hessians who came to this country during the Revolutionary war, and remained at its close. He

Carsten Niebuhr, the oriental traveller, father of our beloved historian and statesman, furnishes a striking example of the revived recollection of scenes and events long past. When old and blind, and so feeble that he had barely strength to be borne from his bed to his chair, the dim remembrance of his early adventures thronged before his memory with such vividness, they painted themselves as pictures upon his sightless eye-balls. As he lay upon his bed, pictures of the gorgeous Orient flashed upon his darkness as distinctly as if he had just closed his eyes to shut them out for an instant. The cloudless blue of the eastern heavens bending by day over the broad deserts, and studded by night with eastern constellations, shone as vividly before him, after the lapse of half a century, as they did upon the first Chaldean shepherds. And he discoursed with strange and thrilling eloquence upon those scenes which thus in the hours of stillness and darkness were reflected upon his inmost soul.

The case of Tennent, a well known American clergyman of the last century, opens up many interesting trains of thought; but none so worthy of consideration as that of the sudden revival of recollection. He was attacked by a dangerous illness, occasioned, apparently, by severe and protracted study. One morning, after his life had been despaired of, while conversing in Latin with his brother, he suddenly became insensible, and to all appearance dead. His funeral was appointed after the usual interval. But his physician, who was an intimate friend, refused to believe he could be dead; whose conviction was somewhat supported by the averment of one of the persons who assisted in laying out the body, that he thought he had perceived a slight warmth in the region of the heart. So earnest was the physician, that the funeral was postponed; the time was again appointed, and again and again the friend pleaded for a little delay—first an hour, then half an hour, then a quarter—but still no signs of life appeared, and it was determined that the ceremony should proceed. But just at the married an American woman and spent his days here, having acquired our language, but in his old age and feeble state the latter years of his life were apparently obliterated, and his earlier days and his mother tongue revived. What made the case still more remarkable was, that upon partial recovery from his illness, he commenced talking in English again. (Ed. Obs.)

supreme moment, the sunken eyelids were raised for an instant, and the body became once more an apparent corpse. An hour passed away, and another groan was heard, and again the body sunk in apparent death. Another hour and another groan, followed now by slight tokens of returning life. The feeble spark was carefully tended, and the patient was slowly restored to health. But it was apparent that his memory was a complete blank. The past was as entirely forgotten as though he had drank of the waters of Lethe. One day seeing his sister reading, he asked her what it was that she held in her hand. On being answered that it was the Bible, he rejoined, "What is the Bible? I do not know what you mean." In every respect, as far as acquired knowledge was concerned, he was a child again. Slowly and laboriously he recommenced his education, beginning at the simplest rudiments. He was one day reading an elementary Latin book with the brother with whom he was speaking in that language at the time of his apparent decease, when all at once he stopped as though he had received a sudden shock, and declared the book seemed familiar to him. In a short time the veil was entirely lifted, and his past acquirements and experience became once more portions of his conscious being. During all this time he uniformly asserted, that he had the most intense and vivid recollection of all that transpired during the days of apparent, or as he firmly believed, real death. He dared not, he said, relate fully what he had seen in that spirit land; but an account of it would be found among his papers after his decease. That event, however, took place during the disturbance of the war of the American Revolution, and these papers, by a series of singular accidents, were lost, before falling into the hands of his executor, and so were never examined. But if his own testimony—the testimony of a gentleman of unimpeached veracity, who for more than half a century thereafter maintained a character of remarkable soberness and circumspection—is to be relied upon, his soul passed from the body, and entered the world of spirits, where he stood in the full presence of that ineffable glory upon which no man may look and live. Did he, in fact, pass those viewless portals, which, we are told, deny all return? Was his call to life a new birth from the dead? Who knows?

Whatever may be the bearings of this case of Tennent upon the subject of dreams and trances, or apparent death, it is certain that a forgetfulness apparently as absolute as can be conceived, was in fact only apparent; that the light from his past existence was invisible only because obscured by the brighter light from the spirit land; just as the faint stars are invisible when concealed by the obscuring daylight, and wait to be revealed when that shall be withdrawn. It is one of those numerous instances which go far towards warranting the belief that there is no such thing as absolute forgetfulness; that every impression made upon the mind is ineffaceable, every inscription incapable of obliteration. A veil may be drawn between the after-consciousness and the inscription; the characters may be filled up; but this veil is ready at any moment to be withdrawn, the filling up to fall away, when the characters will become as legible as when first traced.

There is another well authenticated case, in some respects still more striking, showing as it does, how slight may be the impressions made upon the mind, which shall yet prove ineffaceable. A poor servant girl, in a German town, was attacked by a violent fever. She was unable to read or write, but during the paroxysms of her disease, she became possessed —so the priests say—by a very polyglot devil. She would keep spouting forth in a loud and monotonous voice, unconnected sentences of Latin, Greek and Hebrew. Sheet after sheet of these ravings were taken down; but those who attempted to find the elucidation of some deep mysteries in the Babel of unknown tongues, got their labor for their pains. At length her physician determined to trace out her antecedents. He succeeded in ascertaining that, many years before, while a mere child, she had been employed as a servant by a learned ecclesiastic, whose habit it was to pace up and down a passage in his house, communicating with the kitchen, and read aloud his favorite books. These scattered and unconnected phrases, caught in the intervals of her labor, were now reproduced by her, after an interval of many years. Passage after passage of the notes taken down from her feverish lips, were identified among the old priest's favorite authors; so that not the least doubt remained as to the origin of the girl's "possessions."

Coleridge, in speaking of this case, adds to it one of the weightiest comments ever uttered. "This instance," he says, "contributes to make it probable, that all thoughts are in themselves imperishable; and that if the intelligent faculty should be rendered more comprehensive," (and that this is probable, the instance cited above from the "Opium-eater," shows conclusively,) "it would require only a different and apportioned organization—the body celestial instead of the body terrestrial —to bring before every human soul the collective experience of his whole past existence. And this, perchance, is the dread Book of Judgment, in whose mysterious hieroglyphics every idle word is recorded. Yes, in the very nature of a living spirit it may be more possible that heaven and earth should pass away, than that a single act, a single thought, should be loosened or lost from that living chain of causes, to all whose links, conscious or unconscious, the free will—our own absolute self—is co-extensive and co-present."

It is no idle question, "Do we ever forget?"

